# P53 together with ferroptosis: a promising strategy leaving cancer cells without escape: Mechanisms and clinical prospects of p53-induced ferroptosis

**DOI:** 10.3724/abbs.2023270

**Published:** 2023-12-09

**Authors:** Jianhao Zhan, Jisheng Wang, Yuqing Liang, Xiaoping Zeng, Enliang Li, Hongmei Wang

**Affiliations:** 1 Department of General Surgery Second Affiliated Hospital of Nanchang University Nanchang 330006 China; 2 HuanKui Academy Nanchang University Nanchang 330006 China; 3 School of Basic Medical Sciences Nanchang University Nanchang 330006 China; 4 Medical College Jinhua Polytechnic Jinhua 321017 China

**Keywords:** p53, ferroptosis, lipid peroxidation, ferroptosis signaling, cancer therapy

## Abstract

TP53, functioning as the keeper of the genome, assumes a pivotal function in the inhibition of tumorigenesis. Recent studies have revealed that p53 regulates ferroptosis pathways within tumor cells and is closely related to tumorigenesis. Therefore, we summarize the pathways and mechanisms by which p53 regulates ferroptosis and identify a series of upstream and downstream molecules involved in this process. Furthermore, we construct a p53-ferroptosis network centered on p53. Finally, we present the progress of drugs to prevent wild-type p53 (wtp53) degeneration and restore wtp53, highlighting the deficiencies of drug development and the prospects for p53 in cancer treatment. These findings provide novel strategies and directions for future cancer therapy.

## Introduction

Tumorigenesis is a multifaceted process in which cell death plays a pivotal role. Cancer cells often exhibit an imbalance between the processes of cell division and apoptosis, as cells that should undergo programmed death do not receive the appropriate signals, leading to uncontrolled proliferation and immortality. Despite extensive research efforts, the underlying mechanisms of this phenomenon remain elusive, resulting in a lack of targeted therapies for cancer treatment. Therefore, it is challenging to identify novel molecules or pathways involved in tumorigenesis.

Traditionally, two types of cell death mechanisms have been recognized: apoptosis (programmed cell death) and necrosis. However, further research has revealed additional forms of cell death mechanisms. In 2012, Dixon *et al*. [1] introduced the concept that ferroptosis constitutes a distinct type of controlled cell death that arises in response to oxidative stress characterized by disruption and coagulation of the mitochondrial outer membrane and lipid peroxide accumulation within the cell membranes [ 2 ‒ 4]. Multiple contemporary investigations have confirmed the existence and significance of this mode. Moreover, several recent studies have demonstrated that ferroptosis is an adaptive process closely associated with the occurrence and progression of cancer [5].

TP53, known as the “guardian of the genome”, has been extensively investigated since its discovery [6]. Moreover, it is critical for tumor suppression [ 7, 8]. Mutations in the gene encoding p53 are frequently observed in human cancers [9]. The scientific community agrees that the ability of p53 to induce apoptosis is essential for its tumor suppressor function. Additionally, p53 is an essential regulator of various pathways, including metabolism and ferroptosis, which are crucial for tumorigenesis.

**Figure 1:**
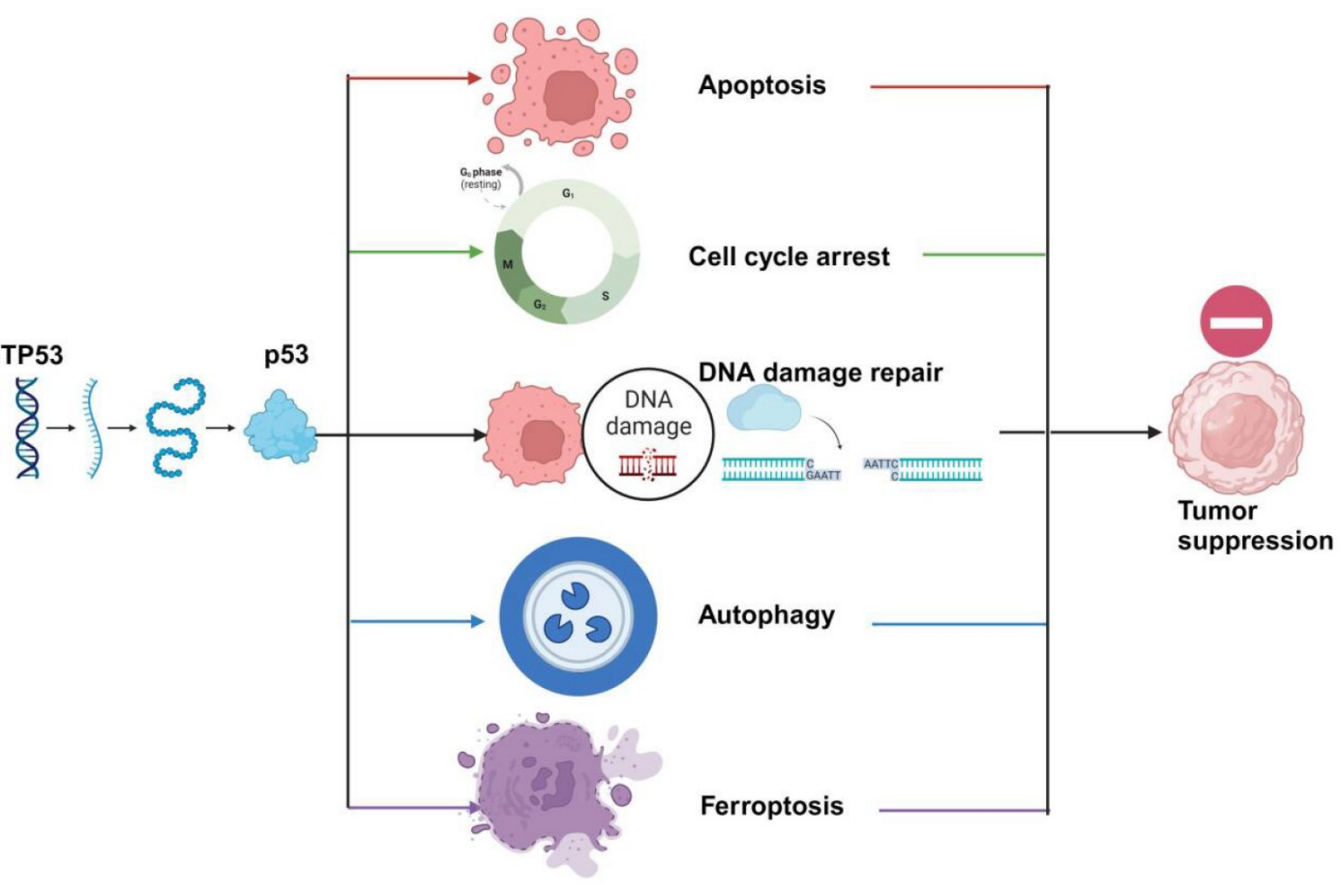
Figure 1 Mechanisms by which p53 suppresses tumors After being activated by stress signals, p53 can regulate downstream target genes to regulate various cell biological processes, including apoptosis, autophagy, cycle arrest, ferroptosis, and DNA damage and repair. Dysregulation of these signaling pathways significantly contributes to tumorigenesis.

P53 mainly exerts its tumor suppressor function by inducing cell senescence, apoptosis, cell cycle arrest, DNA damage repair, and autophagy [ 10‒ 13] ( Figure 1). However, recent studies have revealed that ferroptosis is essential for p53-mediated tumor suppression. Herein, we provide a concise overview of the relevant molecular entities and pathways involved in the tumor-suppressive function of p53. Then, we summarize the mechanisms of p53-regulated ferroptosis, providing new strategies and directions for cancer therapy.

## The Functional Mechanism of P53-suppressing Tumors

Currently, the main method by which p53 suppresses tumors is to induce senescence and apoptosis. P53 induces apoptosis through the B-cell lymphoma-2 (BCL-2) protein family. The induction of BH3-only binding proteins, including recombinant p53 upregulated modulator of apoptosis (PUMA), phorbol-12-myristate-13-acetate-induced protein 1 (PMAIP1) , and Bcl-2 interacting mediator of cell death (BIM) [14], leads to the activation of BCL-2. Consequently, inhibiting the BCL-2 family that promotes survival, including B-cell lymphoma-extra large (BCL-XL), BCL-2, and myeloid cell leukemia-1 (MCL-1), inhibits apoptosis effectors. It releases recombinant Bcl-2 associated X protein (Bax) and recombinant Bcl2 antagonist/killer (BAK) in large quantities [15], increasing mitochondrial outer membrane permeability and cytochrome c release [ 16, 17] and activating cysteinyl aspartate specific proteinase-9 (CASPASE-9), which binds to apoptotic protease activating factor-1 (APAF-1) to form apoptotic bodies [18]. Apoptosome formation activates caspase effectors, ultimately leading to apoptosis [19].

Additionally, p53 suppresses tumors by inducing cell cycle arrest, a mechanism closely related to the wild-type p53 activated fragment-1 (WAF1) gene [20]. The p53 binding site is located 2.4 kb upstream of the WAF1 coding sequence and is particularly closely related to a site on the WAF1 promoter [21]. The specific mechanism is that the p53 gene upregulates cdk inhibitor Cdkn1a (p21CIP1) and p27 kinase inhibitor protein (p27KIP1) (inhibitory cell cycle regulators), which bind to and inhibit Cyclin D1, Cyclin-dependent kinase 4/6 (CDK4/6), and proliferating cell nuclear antigen (PCNA), inhibit DNA replication, and lead to cell cycle arrest [ 22 , 23]. The simultaneous up-regulation of p21CIP1 and p27KIP1 also inhibits Cyclin D1, subsequently promoting the combination of retinoblastoma protein (PRB) and E2F transcription factor 1 (E2F1) and inhibiting DNA replication and E2F1 transcription, ultimately resulting in cell cycle arrest [24].

Tumor cells can invade and metastasize after epithelial-mesenchymal transition (EMT) [25]. The loss of p53 first leads to the establishment of cancer cells after the tumor acquires invasive properties. This loss enables the tumor to penetrate the extracellular matrix (ECM) [26], undergo EMT, and acquire metastatic properties. Tumors that exhibit invasiveness possess the ability to infiltrate capillaries and establish themselves in secondary locations, where they are capable of sustaining their growth and survival [27]. According to research on tumor movement and invasion caused by p53 deletion, p53 can inhibit tumor invasion and metastasis [28]. Matrix metalloproteinases (MMPs) can significantly disrupt the cell basement membrane and ECM [29]. Moreover, p53 has a significant downregulating and inhibitory effect on MMPs [30].

**Figure 2:**
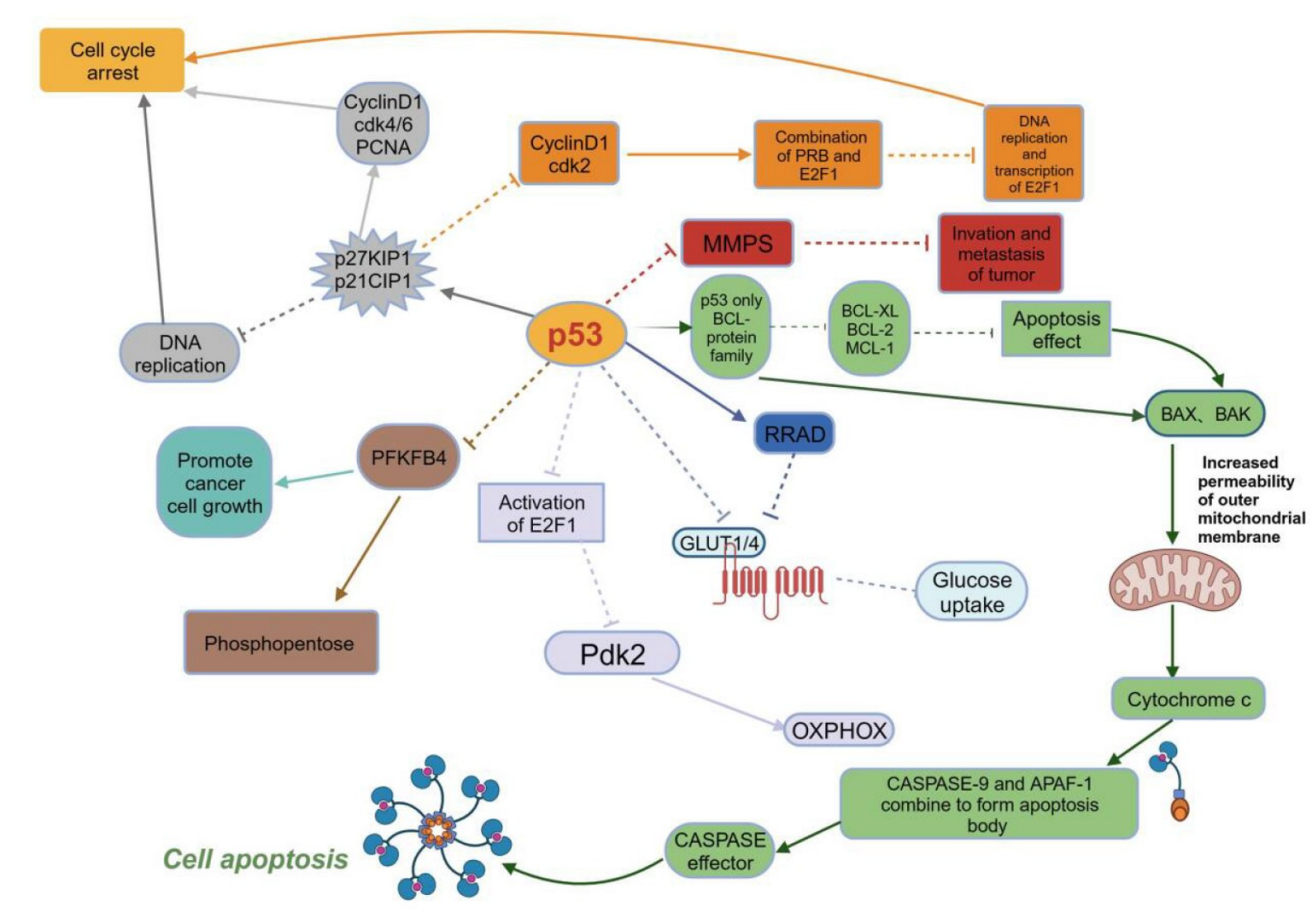
Figure 2 The functional mechanism of p53 suppression of tumors P53 induces BH3-only-binding proteins, leading to apoptosis. To cause cell cycle arrest, p53 inhibits DNA replication and binding and inhibits Cyclin D1, CDK4/6, and PCNA. Upregulation of p21CIP1 and p27KIP1 can also inhibit Cyclin D1, inhibit E2F1 transcription, and achieve cell cycle arrest. P53 can inhibit GLUT1/4 to inhibit glucose uptake. Furthermore, p53 downregulates PFKFB4 and inhibits glucose introduction into the pentose phosphate pathway and glucose metabolism. They all play a tumor-inhibitory role through p53. T-arrows: negative regulation; Solid arrows: positive regulation.

Tumor cells supply the energy required for life activities via glycolysis rather than oxidative phosphorylation (OXPHOX) [31]. P53 may suppress tumor growth by inhibiting glycolysis and promoting OXPHOX. The p53 gene, which inhibits glycolysis, can inhibit glucose transporter 1/4 (GLUT1/4) in a tissue-specific manner to inhibit glucose uptake [32]. Simultaneously, the indirect gene p53 promotes Ras-related associated with diabetes (RRAD) expression, represses GLUT1 [32], and downregulates 6-phosphofructo-2-kinase/fructose-2,6-biphosphatase 4 (PFKFB4). Furthermore, PFKFB4 directs glucose to the pentose phosphate pathway to inhibit glucose metabolism [33]. Cancer cell growth is inhibited by non-expression of PFKFB4 [34]. Therefore, its downregulation suppresses tumor growth. Concurrently, by promoting oxidative phosphorylation, p53 can inhibit E2F activation by inhibiting pyruvate dehydrogenase kinase isoenzyme 2 (Pdk2) and promoting OXPHOX [35]. Nevertheless, further examination is required to determine whether p53 exclusively contributes to the promotion of glycolysis, as certain investigations have indicated its role in the upregulation of hexokinase-2, a pivotal enzyme in glycolysis [36] ( Figure 2).

## The Regulatory Mechanism of P53 in Ferroptosis

**Figure 3:**
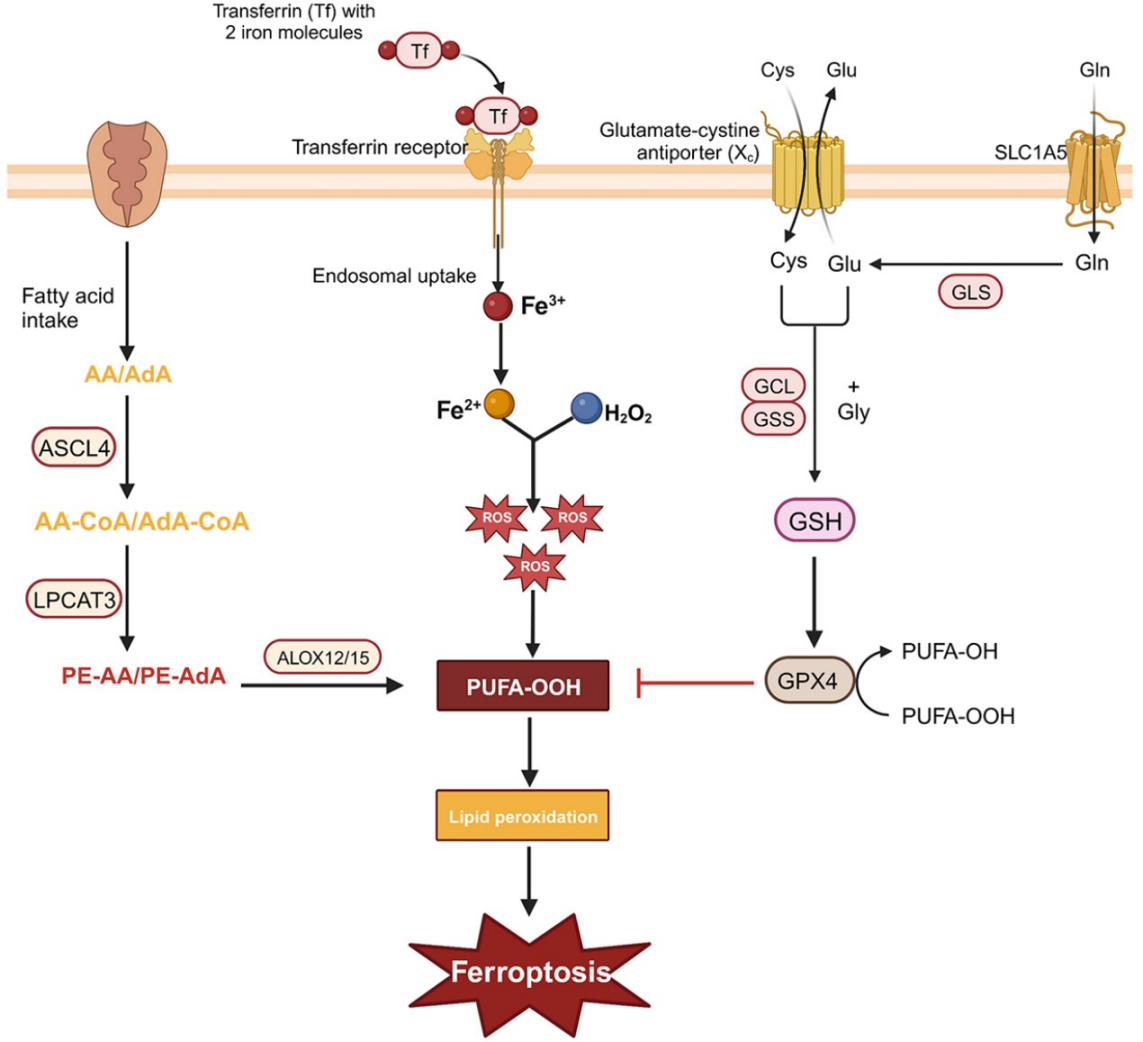
Figure 3 Mechanisms and pathways of ferroptosis The interaction between the oxidative system and the antioxidative system plays a crucial role in the development of ferroptosis. The oxidation system transfers iron from the outside of the cell membrane into the cell through transferrin on the cell membrane. Intracellular iron overload leads to the accumulation of ROS, and then peroxidize the polyunsaturated fatty acids on the cell membrane, which destroy cell membrane and trigger ferroptosis. The antioxidant system transfers cystine outside the cell membrane into the cell to synthesize glutathione through the system Xc – on the cell membrane. Under the catalysis of GPX4, glutathione can remove ROS in cells and inhibit ferroptosis.

Ferroptosis is a distinct form of cell death that occurs due to depletion of the intracellular antioxidant glutathione (GSH), resulting in ROS accumulation and peroxidation of phospholipidated polyunsaturated fatty acids, which ultimately leads to damage and dysfunction of the cell membrane [37]. The intracellular antioxidant system is crucial in ferroptosis, with glutathione peroxidase (GPX4) considered its core regulator [38]. GPX4 is a selenoprotein whose activity is based on the presence of GSH [39]. The two primary sources of cysteine for synthesizing GSH are the glutamate-cystine antiporter (also known as the system X _c_^–^) [40] and the transsulfuration pathway [41]. The system X _c_^–^, composed of SLC3A2 and SLC7A11 subunits connected by disulfide bonds, facilitates extracellular cystine entry into cells while reversing glutamate efflux [42]. After entering the cell, cystine undergoes cysteine by NADPH to participate in GSH synthesis [42]. The transsulfuration pathway involves the interconversion between cysteine and homocysteine [43]. Intracellular oxidative damage and antioxidant defense play a key role in the regulation of ferroptosis ( Figure 3).

### P53 exerts bidirectional regulation on ferroptosis

The activation of the system X _c_^–^ is crucial for resisting ferroptosis, with SLC7A11 serving as a key component of this system. Notably, overexpression of the cystine-glutamate transporter SLC7A11 has been observed in various human cancers [44]. A recent study demonstrated that p53 can inhibit SLC7A11 expression through a transcription-dependent mechanism, reducing cystine intake and inducing ferroptosis in cancer cells [44]. Interestingly, mutants lacking acetylation of p53 ^3KR^ lose their ability to induce apoptosis and senescence but retain the ability to suppress the expression of the SLC7A11 gene [45]. Therefore, tumor cells with mutants deficient in p53 ^3KR^ acetylation cannot trigger senescence and apoptosis. However, they can inhibit tumors by inducing ferroptosis.

Conversely, although the K98 acetylation deletion mutant retains its ability to bind to the SLC7A11 promoter, it loses its capacity to inhibit SLC7A11 expression. Consequently, it cannot impede tumor progression [45]. In addition to directly suppressing SLC7A11 expression, p53 can activate spermidine/spermine acetyltransferase (SAT1) and induce ferroptosis [46]. SAT1 is a rate-limiting polyamine catabolism enzyme critical to converting spermidine and spermine into putrescine [47]. Dysregulation of polyamine metabolism is a common hallmark of cancer [48]. P53 activates SAT1 and induces lipid peroxidation, triggering ferroptosis by inhibiting ROS-mediated tumor growth [46]. Interestingly, induction of SAT1 significantly upregulates arachidonic acid lipoxygenase 15 (ALOX15), which is essential for oxidative stress-induced cell death [49]. However, activating SAT1 has a nonsignificant impact on SLC7A11 and GPX4 expression levels [46]. Subsequently, ALOX15-mediated lipid peroxidation exerts a greater influence on SAT1-induced ferroptosis than inhibiting antioxidant systems. However, other studies have shown that in kidney and T lymphocytes, the inhibition of GPX4 is more significant than the activation of ALOX15 to induce ferroptosis [ 50, 51]. The mechanism that dominates the induction of ferroptosis varies between different cell types. Therefore, separate discussions are required when evaluating their sensitivity to this process.

In certain contexts, p53 may exert opposing effects on ferroptosis. In contrast to its typical role in downregulating SLC7A11 and inducing ferroptosis in various cell types, p53 exhibits a distinct behavior in vascular smooth muscle cells (VSMCs). Specifically, it upregulates the expression of SLC7A11 and inhibits the occurrence of ferroptosis in VSMCs [52]. Additionally, p53 can inhibit ferroptosis in colorectal cancer cells through a transcription-independent mechanism [53]. The DPP4 protein (dipeptidyl peptidase-4) is crucial in mediating cell death. Moreover, DPP4 enzyme activity is closely associated with tumor severity [54]. DPP4 is found in the plasma membrane and forms a complex with NADPH oxidase 1 (NOX1) to facilitate ferroptosis. A recent study revealed that p53 promotes the localization of DPP4 in an inactive ribozyme pool and forms a DPP4-p53 complex to inhibit erastin-induced ferroptosis in colorectal cancer cells [53]. This discovery has revealed the contrasting roles of p53 in ferroptosis between colon and non-colon cancer cells.

Furthermore, researchers have observed that stabilization of wtp53 can delay cystine deprivation-induced ferroptosis in human HT-180 fibrosarcoma cells [55]. This delayed effect depends on the involvement of the p53 transcriptional target (encoding p21) and is associated with decelerated intracellular GSH depletion and reduced accumulation of nuclear ROS [56]. Through inhibiting Cyclin-dependent kinases (CDKs), p21 can induce cell cycle arrest [56]. The stabilization of p53 may reduce the sensitivity of cells to system X _c_^–^ inhibition-mediated ferroptosis by inhibiting its activity. This process may require p21-dependent inhibition of other or alternative CDKs, which can preserve GSH and suppress ferroptosis by inducing complete cell cycle arrest.

Bidirectional regulation of ferroptosis by p53 can be attributed to its ability to inhibit the system X _c_^–^ through different mechanisms. Although acetylation-deficient mutants of p53 ^3KR^ directly promote ferroptosis, wtp53 inhibits ferroptosis by activating p21 and indirectly suppressing the system X _c_^–^ [ 53, 55]. Although both forms of p53 inhibit the activity of the system X _c_^–^, their opposing regulatory effects on ferroptosis highlight the complexity of this pathway. The crucial point is that wtp53 can activate p21, while mutants deficient in acetylation lack this capability. One possible explanation for this inconsistency is that the downregulation of system X _c_^–^ activity and upregulation of p21 may represent two coordinated branches of p53-mediated responses. This coordination is regulated by various post-translational modifications, including phosphorylation and acetylation, which render cells differentially susceptible to ferroptosis in specific contexts. P53 regulates ferroptosis and has a bidirectional effect on many other biological processes, including autophagy, metabolism, oxidation, and anti-oxidation. This complexity manifests in its role in tumor suppression [57], allowing cells to make different adaptations under varying environmental pressures and enabling them to adapt to their surroundings. Moreover, the utilization of diverse cell types in various studies results in distinct genome structures, expression profiles, and signaling pathways. Consequently, while p53 represses SLC7A11 expression in most cells [44], it promotes its expression in VSMCs [52].

### P53 executes ferroptosis in a GPX4-independent manner

The role of GPX4 in ferroptosis is of significant importance, as it employs GSH to directly catalyze the reduction of hydrogen peroxide phospholipids into hydroxy phospholipids. Consequently, GPX4 acts as an inhibitor of ferroptosis within cancer cells [38]. Nevertheless, p53-mediated ferroptosis is independent of GPX4 activity. As previously mentioned, during SAT1-induced ferroptosis, no significant change was observed in the GPX4 expression level. However, the lipoxygenase ALOX15 expression level was significantly increased [46]. In diffuse large B-cell lymphoma (DLBCL), APR-246 can convert mutant TP53 to wild-type TP53 and induce TP53 mutant ferroptosis. The results showed that in this p53-ferroptosis model, the GPX4, SLC7A11, ACSL4, and TfR1 levels did not change significantly. However, ALOX5, ALOX12, and ALOX15 expression levels were significantly increased. Therefore, the p53-ferroptosis pathway triggered by APR-246 is also independent of GPX4 activity [58]. Additionally, while p53 inhibits the expression of SLC7A11 and consequently suppresses cystine uptake, it has minimal influence on the GSH/GSS ratio, the overall level of GSH, and the downregulation of GPX4. Instead, by inhibiting SLC7A11, ALOX12 is indirectly activated to induce ALOX12-dependent ferroptosis under ROS stress. Notably, ALOX12 is essential in p53-mediated ferroptosis [59]. Both studies have demonstrated that the expression level of GPX4 remains significantly unchanged in p53-mediated ferroptosis, indicating its dispensability in this process. Conversely, the levels of the related lipoxygenases ALOX12 and ALOX15 were increased, suggesting their crucial role in mediating membrane oxidative damage.

A recent study has also concluded that Ca ^2+^-independent phospholipase A2β (IPLA2β) is a calcium-dependent phospholipase responsible for hydrolyzing oxidized cardiolipin and releasing oxidized fatty acids [60]. Chen *et al*. [61] emphasized that p53 can regulate ferroptosis by controlling IPLA2β independent of GPX4. Similar to GPX4, IPLA2β plays a role in the regulation of ferroptosis by employing distinct mechanisms to detoxify lipid peroxides. Conversely, GPX4 inhibits ferroptosis by converting oxidized phospholipids into non-toxic lipid alcohols. IPLA2β protects cells from ferroptosis by cleaving peroxidized lipids for detoxification. Unlike GPX4, the loss of IPLA2β does not impact cell growth, development, or viability in normal tissues due to its unique properties. Therefore, IPLA2β is expected to be a novel therapeutic target [61] .

Why is the activity of GPX4, a key enzyme in the antioxidant system, less important than the activation of the oxidative system in p53-induced ferroptosis? Here are some possible explanations. GPX4 is a selenoproteinase that catalyzes intracellular GSH to reduce peroxidized membrane lipids. Therefore, the function of GPX4 depends on GSH levels [38]. However, in the case of p53-induced ferroptosis, while inhibiting SLC7A11 expression can reduce GSH synthesis, activation of other downstream targets of p53 can increase GSH levels. For example, TP53-induced glycolysis and apoptosis regulator (TIGAR) activation by p53 and conservation of GSH through p21 neutralization contribute to maintaining a relatively stable GSH level [ 55, 62]. This is one reason GPX4 activity does not play a significant role in p53-induced ferroptosis. Most current research on p53-induced ferroptosis targets also focuses on activating the lipid oxidation system. For instance, SAT1, a direct downstream target of p53, can enhance ALOX15 expression and activate the oxidation system [46]. Although p53 inhibits SLC7A11 expression, subsequent studies have shown that SLC7A11 can specifically bind to ALOX12 and inhibit its activity. Hence, the inhibition of SLC7A11 expression by p53 leads to the indirect activation of ALOX12, thereby facilitating the oxidative system [59]. Moreover, downstream targets of p53, including IPLA2β, exhibit antioxidant properties and can replace GPX4 to induce ferroptosis in a GPX4-independent manner. Therefore, for p53-regulated ferroptosis, promoting the lipid peroxidation system is more dominant than inhibiting the antioxidant defense system. There appear to be additional mechanisms underlying p53-induced ferroptosis, which differ from the currently recognized GPX4 model. Here, we divide p53-induced ferroptosis into classic GPX4-dependent and non-classical GPX4-independent models for discussion. Since GPX4 activity largely depends on intracellular GSH level, the classical GPX4 model is mainly regulated by the p53/SLC7A11/GPX4 axis, and p53 acetylation plays an important role in this process. Compared to the classical GPX4 model, the non-classical p53-ferroptosis model is more dependent on the activity of ALOXs. The p53/SLC7A11/ALOX12 [59], p53/SAT1/ALOX15 [46], and APR-246/p53/ALOX axes belong to this model. Throughout the p53/IPLA2β axis, the detoxification function executed by IPLA2β demonstrates adequacy in substituting GPX4.

Interestingly, IPLA2β was activated by p53 in the early stages of stress; however, this activation disappeared in the late stages of stress [61]. This regulation can improve the ability of our bodies to adapt to the surrounding environment. In instances of reduced oxidative stress, p53 can induce the activation of IPLA2β, thereby facilitating the detoxification process and impeding the occurrence of ferroptosis. Conversely, when oxidative stress reaches excessive levels, the aforementioned mechanism is inhibited. The retrieval of peroxidized lipids poses challenges; thus, p53 employs a mechanism involving the inhibition of IPLA2β and the induction of ferroptosis to eliminate impaired cells [61]. Briefly, the mechanism of p53-induced ferroptosis exhibits a high degree of complexity. Additional research is necessary to clarify the underlying mechanisms of concern.

### Regulation of the p53-ferroptosis pathway

In p53-mediated ferroptosis-related pathways, numerous downstream target molecules have been documented. Subsequently, we have summarized the upstream molecules that regulate the p53-ferroptosis pathway, which plays a crucial role in the p53 ferroptosis axis.

#### Indirect regulation of the p53-ferroptosis pathway by post-translational modifications

As mentioned above, acetylation of p53 is critical in p53-induced ferroptosis [45]. P53 inhibits SLC7A11 expression by acetylating its DNA binding domain [ 44, 45]. Therefore, the regulation of p53 acetylation is important for the p53-ferroptosis pathway. CREB-binding protein (CBP) is a key molecule for p53 acetylation. In lung tissue, signal transducer and activator of transcription 6 (STAT6) can bind to CBP and inhibit the activity of CBP, which deacetylates p53 and suppresses its inhibitory effect on SLC7A11 [63]. Furthermore, in cardiomyocytes, ubiquitin-specific protease 22 (USP22) deubiquitinates the NAD-dependent protein deacetylase sirtuin-1 (SIRT1), which consequently deacetylates p53, suppresses the inhibitory effect on SLC7A11 and then inhibits ferroptosis. Through the SIRT1/p53/SLC7A11 axis, USP22 may suppress ferroptosis in cardiomyocytes *in vivo*, thereby playing a crucial role in preventing myocardial ischemia/reperfusion injury [64].

In addition to acetylation, phosphorylation is important in the p53-ferroptosis pathway. Bromodomain-containing protein 7 (BRD7), known as celtix-1, is a tumor suppressor that participates in transcriptional regulation by interacting with acetylated histones on chromosomes [65]. *BRD7* knock-in can increase p53 expression in mitochondria and significantly trigger serine 392 phosphorylation [66], which is essential for the location of mitochondrial ectopic p53 [67]. Mitochondrial translocation of p53 is critical for BRD7-induced ferroptosis in hepatic stellate cells (HSCs). The interaction between mitochondrial p53 and SLC25A28 significantly increases the iron content within mitochondria, disrupting ferroptosis homeostasis in BRD7-enhanced HSCs [66]. Mitochondrial iron accumulation within the BRD7-p53-SLC25A28 axis enhances electron transport chain (ETC) function, leading to lipid peroxidation and, ultimately, ferroptosis [66].

#### Direct regulation of the p53-ferroptosis pathway by proteins

Many intracellular proteins are also involved in the regulation of the p53-ferroptosis network. In colon cancer cells, cytoglobin (CYGB) can upregulate ACSL4 expression through the CYGB/p53/YAP1/ACSL4 axis, causing lipid peroxidation and increasing ferroptosis sensitivity [68]. In osteosarcoma cells, bavachin can promote p53 expression by downregulating STAT3. P53 overexpression can inhibit SLC7A11, thus inducing ferroptosis [69]. Cold-induced RNA-binding protein (CIRBP) can bind to the 3′ untranslated region (3′-UTR) of mRNA to regulate target gene expression [70]. CIRBP can directly bind to p53 RNA in pancreatic cancer, inhibiting p53 translation and inducing ferroptosis. Cold induction increases the expression of CIRBP, suppressing the expressions of p53 and GPX4 while promoting the expressions of DPP4, NOX1, and ferritin heavy chain 1 (FTH1) [71]. Therefore, downregulating CIRBP through the CIRBP/p53/ferroptosis pathway may be an important target for treating pancreatic cancer. Mex-3 RNA binding family member A (MEX3A), another RNA-binding protein, has been implicated in cancer regulation. A recent study revealed that MEX3A promotes ubiquitination and degradation of the wtp53 protein in ovarian cancer. Overexpression of MEX3A induced p53 degradation, which inhibited ferroptosis and promoted tumorigenesis in ovarian cancer. Furthermore, the downregulation of glutaminase 2 (GLS2) and upregulation of SLC7A11 resulted from p53 degradation subsequently inhibit ferroptosis [72]. Interestingly, rather than exerting regulatory control over p53 mRNA, MEX3A facilitates the destabilization of the p53 protein, leading to its degradation via the process of ubiquitination. Furthermore, it should be noted that MEX3A does not exhibit any interaction with mutp53. Only wild-type p53 (wtp53) can undergo regulation in this context [72]. Nevertheless, the mechanism underlying this selectivity remains unclear and requires further investigation.

#### Feedback regulation of the p53-ferroptosis pathway

Additionally, the p53-ferroptosis pathway has feedback regulation. Intracellular iron overload, ROS accumulation, and decreased GSH levels are hallmarks of ferroptosis. Furthermore, the levels of these small molecules produce feedback regulation of p53-induced ferroptosis. For example, people of African descent with the p53 P47S polymorphism have high levels of GSH and CoA, and intracellular GSH and CoA can feed back the activity of p53, further reducing ferroptosis sensitivity. Therefore, people of African descent with the p53 P47S polymorphism are often deficient in ferroptosis [73].

Additionally, intracellular iron metabolism feedback regulates the activity of p53. For example, in a sarcopenia model, intracellular iron overload feedback activates p53, inducing ferroptosis through the p53/SLC7A11 axis [74]. Interestingly, feedback regulation by the intracellular iron content differs in different models. Iron reduction rather than overload in the middle cerebral artery occlusion model activates the p53/SLC7A11 axis to induce iron death and cause cerebral ischemic injury [75].

**Figure 4:**
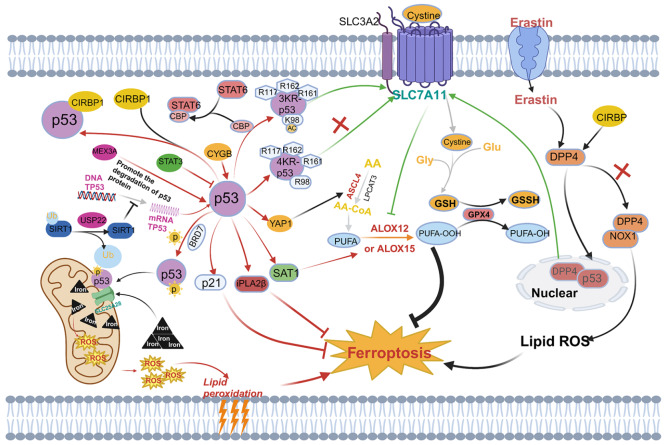
Figure 4 A network of p53-regulated ferroptosis Glutamate-cystine antiporter (system Xc–) can transfer cystine into cells to synthesize GSH. Under GPX4 catalysis, GSH can reduce peroxidized PUFA to inhibit ferroptosis. SLC7A11 is a key component of the system X c–. P53 can promote ferroptosis by inhibiting SLC7A11 and activating SAT1. At the same time, it can also inhibit ferroptosis by binding to DPP4 and inducing p21 expression. Interestingly, p53-mediated ferroptosis does not necessarily depend on GPX4 activity. Compared with the inhibition of GPX4 function, the activation of ALOX12 and ALOX15 is more important. P533KR acetylation-deficient mutants retain the ability to repress SLC7A11 gene expression. However, the K98 acetylation-deficient mutant lost this ability and cannot inhibit tumorigenesis. In addition to downstream molecules, the upstream molecules that regulate p53 (as the figure shows) also play an important role. A series of upstream and downstream p53 molecules were identified to construct a p53-centered ferroptosis regulatory network. T-arrows: negative regulation; Solid arrows: positive regulation.

P53-induced ferroptosis is involved in various biological processes of cells and is closely related to the occurrence and development of cancer. Therefore, the p53-ferroptosis pathway is finely regulated in cells, allowing cells to self-regulate in response to complex external environmental changes. Additionally, the extensive regulatory factors of this pathway provide rich therapeutic targets for the clinical treatment of cancer. Consequently, targeting the p53-ferroptosis pathway has broad clinical prospects ( Figure 4).

## Clinical Prospects of P53 in Cancer

In the previous sections, we briefly described the relevant mechanisms and pathways by which p53 suppresses tumors and focused on downstream target molecules of p53 related to ferroptosis. On this basis, we added upstream-related molecules of the p53-regulated ferroptosis pathway and p53 downstream-related lncRNA molecules, establishing a complete p53-centered ferroptosis network. Research on the mechanism is beneficial to the clinical tumor treatment plan. Next, we elaborate on the clinical application prospects of p53 ( Table 1 ).

**Table:** **Table 1** Drugs under clinical trials for p53-related cancer treatment

Compound		Mechanism		Current progress		Ref.
P53-targeted therapeutic drugsPreventing the degradation of wtp53				
Nutlins		Inhibition of p53 MDM2 binding.		*In vitro* study		[ 76, 77]
Nutlins derivative RG7112 (RO5045337)		It can strongly activate wtp53 in tumors over expressing MDM2, but cannot activate p53 pathway in cancers overexpressing MDMX.		Clinical trial evaluation		[78]
ALRN-6924		MDM2 and MDMX dual inhibitors.		Clinical trial evaluation		[ 79, 80]
Restore the normal function of mutant p53				
p53-n239y or p53-n268d ^a^		Restoration of heat stability and/or sequence specific DNA binding of human p53.		*In vitro* study		[81]
Reduce temperature		Folding of mutant p53.		*In vitro* study		[82]
Some molecular chaperones, such as glycerin		The solvent is directly removed from the vicinity of the protein so that the protein forms more compact folds and is closer to the active conformation.		*In vitro* study		[83]
CP-31398		CP-31398 binds to the denatured DNA-binding domain of mutp53 and restores natural p53 conformation.		*In vitro* study		[84]
APR-246 (methylated analogue of PRIMA-1)		APR-246 enhances wtp53 stability at 37°C, induces a conformational change of mutp53, and restores their DNA binding ability.		Phase I/IIa clinical trial in patients with leukemia, lymphoma, or prostate cancer		[85]
PK083		PK083 binds and stabilizes p53-Y220C to restore its transcriptional activity.		*In vitro* study		[86]
PK7088, PK11000, PK11007, PK11010		Increase thermal stability of mutant p53, thereby restoring wild-type activity.		*In vitro* study		[87]
NSC319726 (ZMC1)		ZMC1 provides an additional Zn ^2+^ to cancer with mutp53 for proper folding.		*In vitro* study		[88]
COTI-2		It has activity on tumor cells carrying mutant TP53 and wild type TP53, and because it inhibits PI3K-AKT pathway.		Phase I trial of gynecological tumors and head and neck cancer		[89]
PTC124 (ataluren)		PTC124 directly acts on firefly luciferase, rather than inducing early termination of codon linkage.		Clinical trial evaluation		[ 90, 91]
Aminoglycoside antibiotics, *e.g.*, G418 (generic) and gentamicin		TP53R213X and other TP53 nonsense mutants can be translated and read through and can induce functional full-length p53.		*In vitro* study		[92]
Aminoglycoside derivative NB124		The nonsense mutations TP53R213X, TP53Q192X, and TP53E298X of TP53 showed the ability to induce reading through.		*In vitro* study		[93]
Suppress mutant p53						
HDACis		It inhibits histone deacetylases (HDACs), thereby regulating gene expression. In addition, HDACis also destroys HDAC6/Hsp90/mutp53 chaperone complex, making the mutp53 protein unstable and promoting its degradation.		*In vitro* study		[ 94, 95]
Hsp90 inhibitor		Targeting another mutp53 protein stabilizer, Hsp90, and inducing apoptosis of p53 deficient cancer cells.		*In vitro* study		[96]
Glycolic acid, traditional Chinese medicine		Promote the degradation of mutp53 proteasome through the carboxyl-terminal of chaperone-associated ubiquitin ligase of Hsp70 interacting protein (CHIP).		*In vitro* study		[97]
Targeted functional pathway of p53 inhibition of tumor				
M6620		Cell cycle regulator ATR inhibitor.		Phase 2 clinical trial		[98]
Treatment of p53-induced ferroptosis inhibiting tumor-related pathways				
Erastin		It can cause ROS production and activate p53, so it can enhance iron death and has specificity for cancer cells.		*In vitro* study		[99]
Levobupivacaine		Inhibit SLC7A11/GPX4 and promote p53 mediated iron death.		*In vitro* study		[100]
NC06		Promote p53 and heme oxygenase-1, thereby reducing iron death caused by oxidized glutathione in colon cancer.		*In vitro* study		[101]
Eupaformosanin		Ubiquitination of p53 and induction of iron death in triple-negative breast cancer.		*In vitro* study		[102]
Flubendazole		Targeting p53 and promoting iron death in castration-resistant prostate cancer.		*In vitro* study		[103]
Disulfiram/copper		Via ROS/MAPK and iron death pathway.		*In vitro* study		[104]
NDV		Promote iron death through the p53-SLC7A11-GPX4 pathway, the release of ferrous ion, and the enhancement of Fenton reaction.		*In vitro* study		[105]
Small molecule MMRi62		Induce iron death and inhibit metastasis of pancreatic cancer by degrading ferritin heavy chain and mutant p53.		*In vitro* study		[106]
Low concentration PTX		Enhance RSL3-induced iron death by up-regulating the expression of mtp53.		*In vitro* study		[107]
Tan IIA		Inhibit the proliferation of gastric cancer by inducing p53 up-regulated iron death.		*In vitro* study		[108]
Nano-PM@CeO2 ^b^		Synergistic iron death.		*In vitro* study		[109]

^a^Introducing the second site to inhibit gene mutation; ^b^Nano-PM@CeO2 is the assembly of p53-activating peptide 2 by CeO2 construction of organometallic supramolecules by nanoparticles.

### P53-targeted therapy

The most immediate treatment is p53-targeted therapy, which includes preventing wtp53 degradation, restoring the wild-type function of mutp53, and inhibiting mutp53.

To prevent the degradation of wtp53, the interaction between p53 and its negative regulator (especially MDM2) is mainly interfered with to prevent subsequent ubiquitination. MDM2 is a main negative regulator of p53 that prevents p53 from entering the nucleus, inhibits p53-DNA binding, and promotes p53 proteasomal degradation [110]. After the discovery of a class of cis-imidazoline analogs (nutlins) that inhibit p53-MDM2 binding, MDM2 inhibitors have been widely investigated as targeted therapies for patients with wtp53 [ 111, 112 ]. Nutlin-3a is a preclinical drug that inhibits tumor growth by reactivating wtp53 alone or in combination with other therapies. This has been demonstrated in lung cancers [76] and prostate cancers [77]. Due to the good results of the *in vitro* studies, a clinical trial was conducted to evaluate the efficacy and safety of the nutlin derivative RG7112 (RO5045337) [78]. Subsequently, most patients treated with RG7112 had stable disease. However, one drawback is that they do not activate the p53 pathway in cancers overexpressing MDMX. Because MDMX had slightly different N-terminal p53 binding pockets, nutlins can strongly activate wtp53 in MDMX overexpressing tumors [113]. ALRN-6924 represents the sole dual inhibitor of MDM2 and MDMX that is currently undergoing clinical trials. Preclinical studies have demonstrated that ALRN-6924 has considerable anti-tumor effects [ 79, 80]. Since MDM2 and MDMX have different anti-p53 activities, dual antagonists targeting p53-MDM2 and p53-MDMX may have a greater inhibitory effect than inhibiting either of them alone.

*TP53*, a tumor suppressor gene, is the most mutated cancer gene [114]. Wtp53 can inhibit tumor development in various ways. However, TP53 mutations and the resulting inactivation of p53 lead to tumor cell death escape and rapid tumor progression [115]. Therefore, clinical efforts to restore the normal function of mutp53 to trigger tumor cell death and eliminate tumors have become a popular research direction for tumor treatment. Accordingly, fixing the tumor suppressor function of p53 may be insufficient to destroy tumors with multiple deficiencies in potently activated cancer drivers, including RAS, PI3K, and MYC. Nevertheless, it can be inferred from the observation that wtp53 is frequently triggered by oncogenic stress that the reactivation of mutp53 within the microenvironment of tumor cells is facilitated by oncogenes that promote growth and the subsequent induction of cancerogenic stress. This ultimately leads to a remarkably effective induction of cell death in tumor cells [ 116‒ 118 ].

First, mutp53 is a viable target. An increasing number of small molecules can advance the correct folding and reactivation of common missense mutp53 proteins. For example, they introduce a second-site suppressor mutation, p53-n268d or p53-n239y, which restores the thermal stability and sequence-specific DNA binding of human p53. The mechanism of this effect was investigated, and both gamma-aminobutyric acid substitutions reduced the free energy of tumor-associated p53-V143A and p53-G245S mutants and restored their ability to bind to target DNA [81]. Studies have shown that lowering the temperature can also lead to the folding of mutp53. The folding of p53 mutants with amino acid substitutions in the β-sheet is temperature-sensitive, such as in human p53-v143a mutants [ 119, 120] and mouse p53-a135v mutants [ 121 , 122]. At 37°C, these mutants are unfolded and non-functional. However, they are folded and retain the functions of wtp53, sequence-specific DNA binding, and transcriptional activation at 32°C [82]. Furthermore, specific molecular chaperones can promote the stability and function of mutp53, including glycerol [83]. The mechanism may be that the solvent is excluded directly from the vicinity of the protein, thereby causing the protein to form tighter folds closer to the active conformation.

Currently, compounds targeting missense mutp53 are being investigated. A growing number of small molecules, including APR-246 (a methylated analog of PRIMA-1), PK11007, CP-31398, PK083, NSC319726 (ZMC1), and folinic acid, can reactivate missense mutp53. These compounds were identified by screening the chemical library, rational drug design, or molecular modeling. They can reactivate missense mutp53 through covalent and non-covalent binding, acting as Zn ^2+^ chelators and disrupting the polymerization of mutp53. Compounds APR-246 and COTI-2 have progressed to clinical trials. The safety of APR-246 has been studied in phase I/IIa clinical trials in patients with lymphoma, leukemia, and prostate cancer, showing a good toxicity profile [85]. The most common ADRs (Adverse Drug Reactions) were headache, fatigue, dizziness, and confusion. However, it is important to note that all negative occurrences observed during the study were ultimately reversible, and no indications of myelotoxicity were detected. Additionally, this study showcased its clinical implications. CT scans displayed reduced bone marrow blast cells in patients with acute myeloid leukemia (AML) and decreased tumor lesions in patients with non-Hodgkin’s lymphoma. Upregulation of pro-apoptotic p53 target genes in patients with leukemia supports antitumor activity [123]. These results and clinical efficacy data from expansion cohorts, including patients with chronic lymphocytic leukemia (CLL) and AML [124], proved that five of six patients with mutant TP53 had some clinical effect. However, not all mutations were missense. Nonsense mutations were also observed (mentioned later). Therefore, the clinical response may be unrelated to the presence of the missense mutation in TP53.

The clinical responses of all wtp53 patients did not meet the predefined response criteria. A phase II proof-of-concept randomized clinical trial is underway in patients with TP53-mutated and recurrent high-grade serous ovarian cancer. The study compared APR-246 with standard carboplatin, pegylated doxorubicin, and the combined use of carboplatin and pegylated doxorubicin. The phase Ib/II trial of APR-246 began in combination with the cytosine analog azacitidine in patients with mutated TP53 and myelodysplastic syndrome (MDS) [125]. In addition, a phase Ib/II clinical study of APR-246 with cisplatin and 5-fluorouracil was initiated to treat esophageal cancer. Since COTI-2 has activity in both tumor cells carrying mutant TP53 and wtp53 and inhibits the PI3K-AKT pathway [89], the exact role of mutp53 reactivation in COTI-2-induced tumor cell death remains unclear. COTI-2 is undergoing phase I trials in gynecologic oncology and head and neck cancer.

TP53 has fewer nonsense mutations than missense mutations but accounts for 10% of cancer TP53 mutations [ 126, 127 ]. Compounds targeting nonsense mutp53 have also been investigated. Identifying oxadiazole PTC124 (ataluren) as a read-through inducer on a cell screen using a luciferase reporter gene allowed the expression of full-length mRNA [128]. Nevertheless, the investigation of PTC124 presents ongoing difficulties. PTC124 exerts its effects by directly targeting firefly luciferase rather than eliciting premature stop codon read-through [129]. A clinical trial using ataluren in cystic fibrosis patients with nonsense mutations in the cystic fibrosis transmembrane conductance regulator gene failed to demonstrate significant clinical efficacy [90]. However, a phase III clinical study of patients with Duchenne muscular dystrophy with nonsense mutations in the dystrophin gene demonstrated that the disease progressed more slowly in a subgroup of patients [91].

Additionally, aminoglycoside antibiotics, including gentamicin and G418 (geneticin), can induce translational read-through of several TP53 nonsense mutants (including TP53R213X) and induce functional full-length p53 [92]. The aminoglycoside derivative NB124 can generate read-through against TP53 nonsense mutations TP53R213X, TP53Q192X, and TP53E298X. In H1299 lung cancer cells carrying the TP53R213X nonsense mutant, the p53 target genes *CDKN1A* and *BAX* induced apoptosis, indicating that the full-length p53 protein is active. NB124 also causes read-through nonsense mutations in the APC tumor suppressor gene [93]. The clinical use of aminoglycosides is limited due to their serious side effects (ototoxicity and nephrotoxicity) [ 130, 131 ]. Efforts are ongoing to identify new potent and less toxic read-through compounds.

The last step is to suppress mutp53, mainly by restricting mutp53 expression or depleting mutp53. The mutp53 expression level can be transcriptionally reduced by histone deacetylase inhibitors (HDACis) [132]. HDACis are antineoplastic drugs that inhibit histone deacetylases (HDACs) that regulate gene expression [94]. HDACis also destroy the HDAC6/Hsp90/mutp53 chaperone complex, destabilizing the mutp53 protein and promoting its degradation [95]. Hsp90 inhibitors target Hsp90, another mutp53 protein stabilizer, to induce apoptosis in p53-deficient cancer cells [96]. Furthermore, some compounds can promote the degradation of mutp53. For example, the traditional Chinese medicine gambolic acid promotes the degradation of the mutp53 proteasome through the C-terminus of chaperone-associated ubiquitin ligase of Hsp70-interacting protein (CHIP) [97]. However, in addition to degrading mutp53, these drugs exhibit various antitumor effects [ 133, 134]. The behavior of p53 depletion in antitumor effects should be further discussed, and drugs that selectively target mutp53 should be screened.

### Targeted functional pathway of p53 inhibition of tumors

In addition to targeting the p53 gene and mutp53 with cell cycle arrest and energy metabolism, endless treatment options can use p53 to suppress tumors. Because of the increased dependence of mutp53 cancers on intra-S and G2 arrest, regulators of the intra-S and G2 checkpoints have been identified, including A.T.R. [135], CHK1 [136], MK2 [137], and Wee1 [138]. Several clinical trials have examined numerous regulatory factor inhibitors. M6620 was the first ATR inhibitor tested in humans. A recent phase 2 clinical trial of M6620 reported that compared to gemcitabine monotherapy (median progression-free survival, 14.7 weeks, 90% confidence interval (CI) 9.7–36.7 weeks), M6620 combined with gemcitabine combination therapy (median progression-free survival, 22.9 weeks, 90% CI 17.9–72.0 weeks) was more effective, indicating that ATR inhibitors could enhance the effects of current chemotherapy [98].

### Treatment of p53-induced ferroptosis inhibits tumor-related pathways

Accordingly, we elaborated on a treatment plan for p53-induced ferroptosis to inhibit tumor-related pathways. As previously stated, activating the p53 gene can induce ferroptosis. Recently, many studies have reported compounds that can suppress tumors through the p53-induced ferroptosis pathway. For example, recent findings suggest that erastin activates p53, enhancing ferroptosis. After erastin treatment of lung cancer A549 cells, p53 transcripts were significantly upregulated, and ROS levels increased significantly. Exposure to erastin significantly affected p53 activation after pretreatment with the ROS scavenger N-acetyl-1-cysteine (NAC). This suggests that p53 activation depends on ROS production induced by erastin exposure. Therefore, we conclude that erastin treatment leads to ROS generation, followed by activation of p53 and subsequent activation of the p53 pathway downstream of p53. This aggravates the key cytotoxic and cytostatic effects of erastin on A549 cells, ultimately leading to ferroptosis. However, this effect of erastin has not been observed in normal lung cells, indicating its specificity for cancer cells [99].

In contrast to its anaesthetic effect, levobupivacaine inhibits SLC7A11/GPX4 and promotes p53-mediated iron death, playing an antitumor role in non-small cell lung cancer [100]. Increased doses of the N-acyl sphingosine amidohydrolase (ASAH2) inhibitor NC06 stimulate p53 and heme oxygenase-1, thus causing ferroptosis in colon cancer by reducing oxidized GSH [101]. Eupaformosanin is a natural product that ubiquitinates p53 and induces ferroptosis in triple-negative breast cancer [102]. Flubendazole elicits potent antitumor effects by targeting p53 and promoting ferroptosis in castration-resistant prostate cancer [103]. Disulfiram/copper induces antitumor activity against nasopharyngeal carcinoma cells and cancer-associated fibroblasts through the ROS/MAPK and ferroptosis pathways [104]. Newcastle disease virus (NDV)-induced ferroptosis acts through the p53-SLC7A11-GPX4 pathway. Concomitantly, intracellular ROS and lipid peroxide levels increase in tumor cells. NDV promotes ferroptosis and induces ferritin phagocytosis to kill tumor cells by releasing ferrous iron and enhancing the Fenton reaction [105]. The small molecule MMRi62 induces ferroptosis and suppresses pancreatic cancer metastasis by degrading the heavy ferritin chain and mutp53 [106]. Low concentrations of PTX enhance RSL3-induced ferroptosis by upregulating mtp53 expression [107]. Moreover, some Chinese herbal medicine components can inhibit tumors via the p53-mediated ferroptosis pathway. For example, tanshinone IIA (Tan I.I.A.), a pharmacologically active ingredient isolated from the rhizome of the Chinese herb Salvia miltiorrhiza, induces p53 upregulation-mediated ferroptosis to inhibit gastric cancer proliferation [108]. Some nanotherapeutics also have improved applications. Recently, a new report demonstrated the effect of CeO2 assembly of p53-activating peptide 2 nanoparticles to construct metal-organic supramolecules on synergistic ferroptosis in tumors [109].

### Difficulties and future of p53 tumor therapy research

There exist numerous potential challenges that may impede the progress of preclinical and clinical advancements in p53 therapeutics and novel anticancer agents. For example, compounds identified in protein-based *in vitro* assays may not have good solubility and hydrophobic properties or may be toxic to cells. Therefore, it is a significant challenge to understand the molecular mechanisms by which certain compounds suppress tumors. However, the results of our existing studies on p53 tumor therapy are encouraging. Therefore, it is expected that the clinical efficacy of p53 therapy will be realized in the future. The potential clinical application of this strategy holds promise for enhancing tumor treatment outcomes and prognosis, thereby exerting a substantial influence on public health.

## Conclusions

Herein, we reviewed the upstream and downstream molecules through which p53 regulates ferroptosis, constructing a p53-ferroptosis network centered on p53. All mechanistic studies eventually serve and can be used clinically. Subsequently, we believe that the clinical prospects of tumor treatment related to the p53-ferroptosis network are broad. In the future, an increasing number of molecules situated both upstream and downstream will likely be discovered, thereby contributing to the progressive expansion and refinement of this intricate network. However, the present advancement in the field of novel anticancer therapeutics is hindered by inadequate solubility and hydrophobic characteristics, as well as potential cytotoxicity. Nevertheless, despite these challenges, we remain optimistic about the potential opportunities it presents. However, the data presented in our study indicate that the current findings pertaining to p53 and the therapeutic approach of targeting p53 ferroptosis in tumor treatment are highly promising. The pursuit of p53 ferroptosis-related tumor therapies is exceptional compared to the existing array of treatment modalities, which offers a promising therapeutic strategy for cancer patients and exerts a substantial influence on public health.
